# Effects of *GBA1* Variants and Prenatal Exposition on the Glucosylsphingosine (Lyso-Gb1) Levels in Gaucher Disease Carriers

**DOI:** 10.3390/ijms252212021

**Published:** 2024-11-08

**Authors:** Paulina Szymańska-Rożek, Patryk Lipiński, Grazina Kleinotiene, Paweł Dubiela, Anna Tylki-Szymańska

**Affiliations:** 1 Faculty of Mathematics, Informatics and Mechanics, University of Warsaw, 02-097 Warsaw, Poland; p.szymanska@gmail.com; 2 Institute of Clinical Sciences, Maria Skłodowska-Curie Medical Academy, 00-136 Warsaw, Poland; 3 Faculty of Medicine, Vilnius University, 03101 Vilnius, Lithuania; grazina.kleinotiene@santa.lt; 4 Department of Regenerative Medicine and Immune Regulation, Medical University of Bialystok, 15-089 Bialystok, Poland; paweldubiela89@gmail.com; 5 Department of Pediatrics, Nutrition and Metabolic Diseases, The Children’s Memorial Health Institute, 04-730 Warsaw, Poland

**Keywords:** Gaucher disease, glucosphingosine, placenta, carrier

## Abstract

Gaucher disease (GD) is a lysosomal lipid storage disorder caused by β-glucocerebrosidase (encoded by *GBA1* gene) activity deficiency, resulting in the accumulation of glucosylceramide (Gb1) and its deacylated metabolite glucosylsphingosine (lyso-Gb1). Lyso-Gb1 has been studied previously and proved to be a sensitive biomarker, distinguishing patients with GD from carriers and healthy subjects. It was shown that its level corresponds with β-glucocerebrosidase activity, thus it remains unknown as to why carriers have slightly higher lyso-Gb1 level than healthy population. This is the first report on lyso-Gb1 levels describing representative cohort of GD carriers. Our data of 48 GD carriers, including three newborns, indicated that there are significant differences in lyso-Gb1 levels between carriers having a GD-affected mother and a healthy mother (11.53 and 8.45, respectively, *p* = 0.00077), and between carriers of the L483P *GBA1* variant and carriers of other *GBA1* pathogenic variants (9.85 and 7.03, respectively, *p* = 0.07). Through analysing our unique data of three newborns whose mothers are patients with GD, we also found that lyso-Gb1 is most probably transferred to the foetus via placenta.

## 1. Introduction

Gaucher disease (GD) is an autosomal recessive lysosomal sphingolipid storage disorder caused by a β-glucocerebrosidase deficiency. The severity of GD reflects functional impairment of the enzyme and *GBA1* variants, and thus GD is classified according to the central nervous system involvement as (i) non-neuronopathic GD type 1 (GD1), and (ii) neuronopathic GD type 2 (acute, GD2) or (ii) type 3 (chronic, GD3) [1,2,3,4]. The most common pathogenic genetic variant in *GBA1* in the Caucasian population is NM_000157.4:p.(Asn409Ser) [N370S], responsible for GD type 1 (GD1); the presence of N370S on one *GBA1* allele is protective of the development of a neurological involvement. Most Polish GD1 patients are found to be heterozygous for N370S and other *GBA1* variants, especially L483P [c.1448T>C, p.(Leu483Pro)] [5]. In all forms of GD, signs and symptoms may include hepatosplenomegaly, thrombocytopenia, anaemia, growth delay in children, and bone involvement [1,2,3,4]. Neurological manifestations are a spectrum from horizontal ophthalmoplegia as the only neurological symptom, to more severe forms with neurological signs including progressive myoclonus epilepsy, cerebellar ataxia or spasticity, dementia in some cases and kyphosis [5]. However, Polish GD type 3 patients (mostly L483P homozygotes) are characterised by an early onset (first 2 years of life) massive hepatosplenomegaly [5,6]. Subtle neurological features, such as supranuclear gaze palsy and a mask-like face, generally appear later. These characteristics is very similar to the Norrbottnian-derived Swedish Gaucher population [5,6].

The definite diagnosis of GD relies on demonstration of a deficient β-glucocerebrosidase activity and confirmation by the identification of biallelic pathogenic variants in *GBA1* [6,7]. As in other diseases, specific biomarkers are desirable and expected to improve diagnostic delay, assess the disease severity, and predict the treatment outcomes. Different molecules were tested and some of them are commonly used, including serum chitotriosidase [7]. Chitotriosidase was the first biomarker used in GD and has been shown to correlate with disease initial severity, its progress and the effectiveness of treatment [8]. However, one the greatest limitations of chitotriosidase as a biomarker of GD is a 24 bp duplication in the *CHIT1* gene which is quite common in Caucasians (about 5% homozygous and 35% heterozygous). In 2010, a newly introduced biomarker—glucosphingosine (lyso-Gb1)—was expected to provide a breakthrough in the lysosomal storage disorders (LSDs) field [9,10]. Glucosylosphingosine was first isolated and characterised in GD spleen by Raghavan et al. in 1974 [10]. It was demonstrated that lyso-Gb1 accumulates in tissues as an easily soluble lipid that exists in the lysosomal system to cause wide-spread pathophysiological effects in GD patients.

Several studies showed differences in lyso-Gb1 level among patients with GD, carriers, and healthy controls as collected in a recent review by Giuffrida et al. [9]. Lyso-Gb1 was found undetectable or occurring at the negligible levels in plasma of healthy subjects [11]. Two independent studies, a prospective by Dekker et al. and a retrospective by Rolfs et al. found that carriers levels of lyso-Gb1 are only slightly elevated as compared to healthy subjects [12,13]. In addition, Rolfs’ study determined a plasma lyso-Gb1 threshold of 12 ng/mL (about 26 nmol/mL), allowing for differentiation between patients with GD from healthy subjects, patients with other LSDs, and GD carriers, reaching 100% sensitivity and specificity of the biomarker [13]. Adding a diagnostic cut-off value of 4 ng/mL for plasma lyso-Gb1, with the same 100% specificity, demonstrating clear and precise ranges for diagnostic purposes was established by Revel-Vilk et al. [8]. This was also confirmed by a recent study showing that carriers of *GBA1* pathogenic variants had higher lyso-Gb1 levels (average 5.8; range 2.5–15.3 ng/mL), compared to those homozygous for wild-type *GBA1* (average 4.9; range 1.5–16 ng/mL) [11].

It was also shown that for the paediatric population, lyso-Gb1 is a reliable marker, significantly increasing in patients with GD (1080.1 ± 739.3 ng/mL) relative to non-GD subjects (the median of 4.9 ± 6.5 ng/mL) [14]. There are no data available so far on lyso-Gb1 levels among newborn carriers.

The aim of the study was to assess whether there is any significant difference in lyso-Gb1 levels between GD carriers having either a GD-affected mother or a healthy mother. Secondly, we asked whether GD carriers having the L483P (previously known as L444P) *GBA1* exhibit different lyso-Gb1 levels than those carrying other (non-L483P) *GBA1* variants. We also checked if our data suggest that lyso-Gb1 level decreases with time.

## 2. Results

The results of lyso-Gb1 measurements are presented in Table 1 and Table 2, along with the age and annotations about the source of the *GBA1* variant inheritance, and whether the individual is the L483P *GBA1* carrier or not.

### 2.1. Comparison of Carriers Divided into Two Groups with Respect to Criterion 1 and Criterion 2

**Figure 1:**
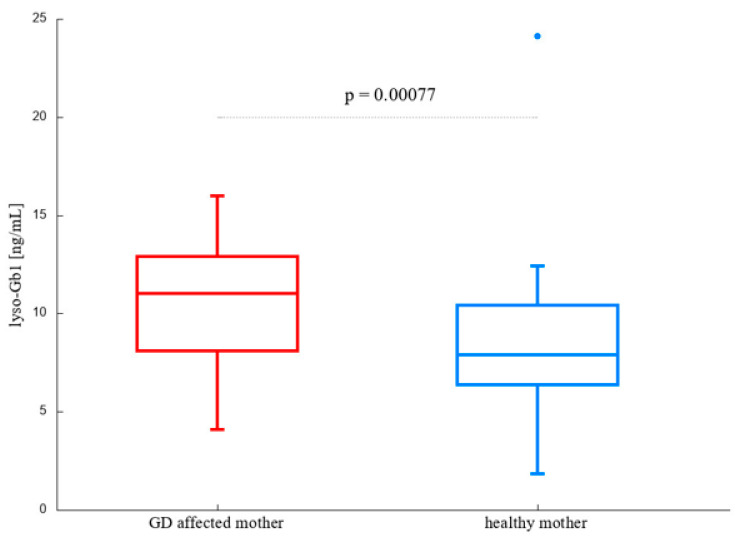
Lyso-Gb1 levels in carriers with affected (n = 19) and healthy (n = 29) mother.

**Figure 2:**
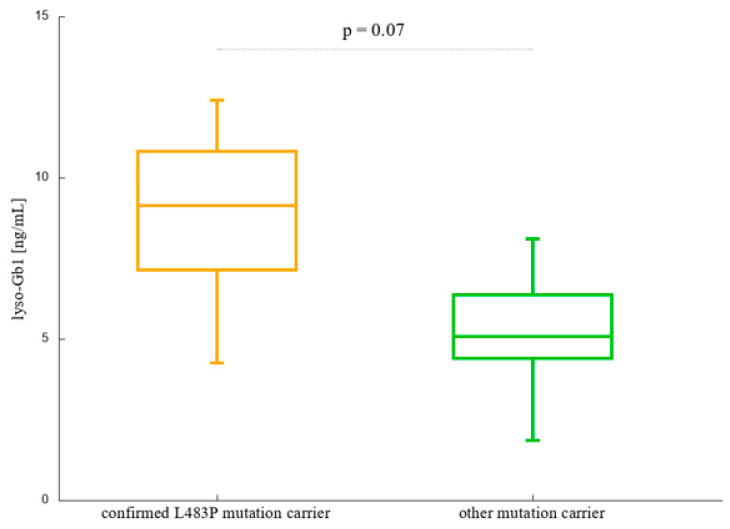
Lyso-Gb1 levels in L483P *GBA1* carriers (n = 16) and other *GBA1* pathogenic variants carriers (n = 13).

We performed two Mann-Whitney U-tests to compare individuals having GD affected mother with those whose mother is not a GD patient, regardless of the fact if she is a *GBA1* pathogenic variant carrier or not (division by criterion 1), and L483P *GBA1* carriers with non-L483P *GBA1* carriers (criterion 2). Considering a 10% level of significance, both criteria turned out to divide the cohort into significantly different groups (*p*-value of 0.00077 and 0.07, for criterion 1 and 2, respectively); however it seems that mother’s status is more likely to divide the carriers into two distinct groups. The results are presented in Figure 1 and Figure 2.

### 2.2. Correlations

**Figure 3:**
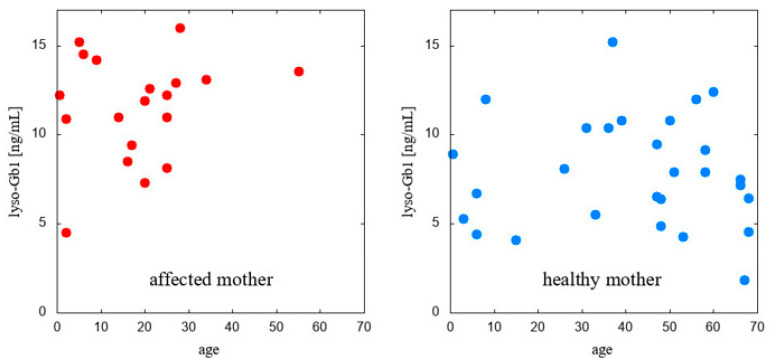
Lyso-Gb1: age correlation in carriers divided with reference to criterion 1.

**Figure 4:**
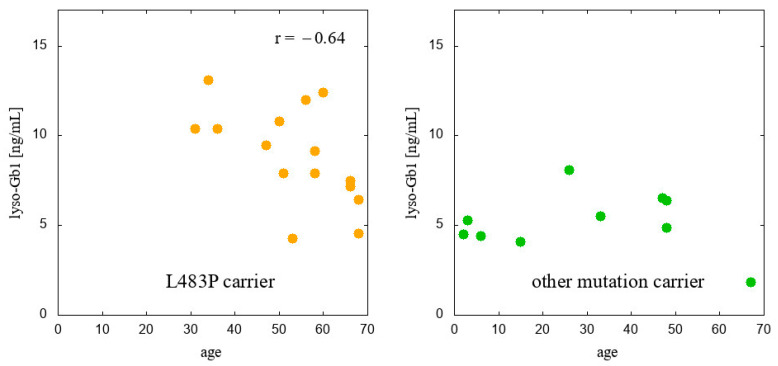
Lyso-Gb1: age correlation in carriers divided with reference to criterion 2.

Calculations of Pearson’s linear correlation coefficients were conducted to examine the relationships between age and lyso-Gb1 levels across specific subpopulations, including *GBA1* carriers with a mother affected by GD, carriers whose mother is not a GD patient, carriers with a confirmed L483P *GBA1* variant, and carriers with a confirmed *GBA1* pathogenic variant other than L483P. The results are presented in Figure 3 and Figure 4.

The only significant correlation occurred in the group of L483P *GBA1* carriers in whom lyso-Gb1 level was found mildly negatively correlated with age (Figure 4).

### 2.3. Mother-Newborn Lyso-Gb1 Dependence

Our unique data of three newborns with lyso-Gb1 measured right after delivery is presented in Table 3. All three newborns were carriers of the non-L483P *GBA1* pathogenic variants. One mother (newborn N1) was not treated with enzyme replacement therapy (ERT; Cerezyme^®^; Genzyme Corporation, Cambridge, MA, USA; 30 U/kg/every other week) and her lyso-Gb1 level was outstandingly high. The other two mothers were treated with ERT. For the individual N1, we also collected the results of lyso-Gb1 levels six months later; it decreased from 77.7 to 12.2 ng/mL. This suggests that the initial high concentration of the biomarker was transferred via the placenta, and quickly decreased; however, slightly elevated lyso-Gb1 persisted regardless of age.

## 3. Discussion

The pivotal role of lyso-Gb1 in pathomechanism, diagnosis, and treatment monitoring of GD has been shown, and thus, is widely applied in clinical practice worldwide [9,10,11,15]. This article provides some new insights regarding lyso-Gb1 in carriers with different types of variants in *GBA1*, highlighting a unique pattern among newborn carriers.

In 2022, Dinur et al. explored the utility of lyso-Gb1 levels in dried blood spot (DBS) samples as a diagnostic marker for GD [11]. Their study highlighted the limitations of using lyso-Gb1 alone to distinguish between heterozygous *GBA1* pathogenic variant carriers and individuals with the homozygous wild-type *GBA1* variant. This conclusion aligns with previous research by Aflaki et al. (2014), which also underscored the challenges of solely relying on lyso-Gb1 for accurate diagnostic differentiation [4]. Notably, the GD diagnosis is commonly established based on β-glucocerebrosidase activity and *GBA1* genotyping. However, the authors called for a paradigm change in the diagnosis of GD based on lyso-Gb1 measurements and confirmatory *GBA1* analyses in DBS.

The placenta plays a critical role in the exchange of various molecules between the maternal and foetal circulatory systems to support foetal development. The transport of disease biomarkers via the placenta is an area of ongoing research. Some studies suggested that certain biomarkers, including those for metabolic, infectious, and genetic conditions, can cross the placental barrier, potentially influencing foetal health or serving as early indicators of a disease [16]. It has been shown that gangliosides pass from the mother through the placenta, which has an impact on the formation and development of the central nervous system (CNS) [2].

It cannot be ruled out that glucosphingosine levels, when increased in the foetal period, may have some effects on CNS formation, not excluding the risk of Parkinson’s disease (PD) in the future [17,18]. However, increased lyso-Gb1 (in carriers from mothers) persists throughout life, even in adulthood, which was demonstrated in our material (Table 1). Our results also emphasised the potential influence of the placental transfer of lyso-Gb1. This implies the necessity of considering placental transfer effects when interpreting lyso-Gb1 levels in newborn screening procedures and in individuals with maternal GD history. Understanding the placental dynamics is crucial, as the transfer of this biomarker might lead to elevated levels in neonates that do not necessarily correlate with their own metabolic status but rather reflect maternal contributions. Our observations are especially important regarding the level of lyso-Gb1 in *GBA1*-associated Parkinson’s disease (*GBA1*-PD), and the fact that an increased risk of developing PD has been observed in both GD patients and carriers [18]. The correlation of several *GBA1* pathogenic variants in GD with the severity of *GBA1*-PD has been shown, i.e., L483P significantly increased PD risk and hastened the disease progression [19,20,21]. L483P *GBA1* carriers exhibited the highest percentage of abnormal clinical tests assessing various domains of PD compared to N370S variant carriers in the study of Becker-Cohen et al. [19]. There is also a link between *GBA1* pathogenic variants (e.g., N370S, L483P) and the accumulation of α-synuclein and formation of its aggregates. In our previous study, the levels of α-synuclein mRNA transcripts were found to be significantly elevated in both GD3 patients and L483P *GBA1* carriers.

On the other hand, besides a genetic factor (*GBA1* pathogenic variant) reflecting lyso-Gb1 level, our study highlights the importance of the second factor, namely transplacental lyso-Gb1 transfer. So far, lyso-Gb3 was found to be accumulated in full-term placentas and umbilical cords of women with Fabry disease [16].

We believe that this analysis is valuable not only for the narrow group of specialists studying GD, but for those who face a broader question of what is more “determinant”—nature (genes) or nurture (foetal environment). In this report, we analysed a large cohort of carriers and found that both the *GBA1* pathogenic variant and mother’s status (whether she is affected or not) are crucial for the biomarker level. The age of the carrier does not seem to play an important role, unless we look at newborns for whom a GD-affected mother transferred the biomarker via placenta. This is the first report reporting lyso-Gb1 level in newborns of GD-affected mothers, ERT treated and untreated. Our study reveals unique lyso-Gb1 patterns among newborn *GBA1* carriers. The potential impact of placental transfer on lyso-Gb1 levels necessitates careful interpretation of analyses performed in newborns and individuals with maternal GD history. Further research should focus on understanding placental transfer mechanisms to enhance the accuracy of lyso-Gb1 assessments, particularly in the context of GBA1-associated Parkinson’s disease.

## 4. Materials and Methods

### 4.1. Patients

A total number of 48 heterozygotic carriers of *GBA1* pathogenic variants were recruited to the study. The whole cohort was divided according to two criteria:

Criterion 1: GBA1 carriers whose mother was a GD patient (19 individuals, median age 20 years, range [0.5;55]) versus carriers with a healthy mother (29 individuals, median age 47 years, range [0.5;68]);

Criterion 2: confirmed carriers of the L483P [NM_000157.4:p.(Leu483Pro)] GBA1 pathogenic variant (16 individuals, median age 54.5 years, range [31;68]) versus con-firmed carriers of other GBA1 variant (13 individuals, median age 25.5 years, range [0.5;67]).

Many of our carriers are so-called obligate carriers, as being parents of our patients with GD, but we do not know from whom those parents received a GBA1 pathogenic variant.

We also disposed the data of three newborns whose mothers are patients with GD. Their lyso-Gb1 levels have been measured straightaway after delivery. These three results were not included in the above-mentioned analysis, however one of them had lyso-Gb1 measured also later; this result is included.

### 4.2. Molecular Analysis

Approximately 60 µL of blood samples were obtained from the veins and spotted on filter paper (DBS, Archimed life Vienna, Wien, Austria); the spots were allowed to dry for 2–4 h at room temperature. Genotyping was performed by a complete gene *GBA1* sequencing [6]. Lyso-Gb1 levels were determined according to the previously described method [10].

### 4.3. Statistical Analysis

The analysis consisted of comparing the means of lyso-Gb1 levels in carriers divided into two groups with respect to criterion 1 (mother’s status) and criterion 2 (mutation variant). The subpopulation of patients with healthy mothers, as well as the subpopulation of carriers having a L438P GBA1 variant did not exhibit normality (*p*-values of Shapiro–Wilk test were 0.0019 and 0.0053, respectively), therefore we performed a non-parametric version of the unpaired *t*-test and Mann–Whitney U test. Simple statistics were calculated, and box and whisker plots constructed. We also verified the hypothesis that lyso-Gb1 level decreases with time by calculation of Pearson’s linear correlation coefficient (age vs. the biomarker level). Calculations were performed in statistical software R 4.3.3.

## Figures and Tables

**Table 1:** Genetic and biochemical characteristics of individuals involved in the study.

Patient Number	Age (Years)	Mutation Inherited from	*GBA1* Variant	Lyso-Gb1 Level (ng/mL) [Reference Value < 12 ng/mL]
1	55	GD Mother	N370S	13.54
2	20	GD Mother	Uncharacterised	11.90
3	25	GD Mother	R48W	12.20
4	25	GD Mother	Uncharacterised	8.13
5	2	GD Mother	Uncharacterised	10.90
6	0.5	GD Mother	N370S	12.20
7	6	GD Mother	Uncharacterised	14.50
8	5	GD Mother	Uncharacterised	15.20
9	16	GD Mother	Uncharacterised	8.50
10	9	GD Mother	Uncharacterised	14.20
11	14	GD Mother	Uncharacterised	11.00
12	21	GD Mother	Uncharacterised	12.60
13	27	GD Mother	Uncharacterised	12.90
14	20	GD Mother	Uncharacterised	7.30
15	25	GD Mother	Uncharacterised	11.0
16	28	GD Mother	Uncharacterised	16.0
17	2	GD Mother	Uncharacterised	4.50
18	17	GD Mother	Uncharacterised	9.40
19	34	GD Mother	confirmed L483P	13.10
20	50	Mother/father carrier	confirmed L483P	10.81
21	66	Mother/father carrier	confirmed L483P	7.15
22	68	Mother/father carrier	confirmed L483P	4.57
23	47	Mother/father carrier	confirmed L483P	9.47
24	68	Mother/father carrier	confirmed L483P	6.45
25	51	Mother/father carrier	confirmed L483P	7.88
26	66	Mother/father carrier	confirmed L483P	7.51
27	53	Mother/father carrier	confirmed L483P	4.26
28	58	Mother/father carrier	confirmed L483P	9.14
29	56	Mother/father carrier	confirmed L483P	11.98
30	58	Mother/father carrier	confirmed L483P	7.90
31	31	Mother/father carrier	confirmed L483P	10.40
32	31	Mother/father carrier	confirmed L483P	24.10
33	60	Mother/father carrier	confirmed L483P	12.40
34	36	Mother/father carrier	confirmed L483P	10.40
35	47	Mother/father carrier	other than L483P	6.54
36	48	Mother/father carrier	other than L483P	4.85
37	48	Mother/father carrier	other than L483P	6.37
38	67	Mother/father carrier	other than L483P	1.85
39	3	Mother/father carrier	T321M	5.30
40	32	Mother/father carrier	T321M	5.50
41	26	Mother/father carrier	T321M	8.10
42	15	Mother/father carrier	R448H	4.10
43	6	GD father	N370S	4.40
44	8	GD father	Uncharacterised	12.00
45	6	GD father	Uncharacterised	6.70
46	37	GD father	Uncharacterised	15.20
47	39	GD father	Uncharacterised	10.80
48	0.5	GD father	N370S	8.90

**Table 2:** Summary of the number of individuals in analysed sub-cohorts.

Individual’s Mother	Confirmed L483P	Other *GBA1* Pathogenic Variant, Confirmed	Uncharacterised *GBA1* Variant
GD mother	1	3	15
Healthy mother	15	10	4

**Table 3:** Lyso-Gb1 levels measured in three newborn carriers of a non-L483P *GBA1* pathogenic variant, measured right after delivery, given along with mother’s results. The norm was <14.0 ng/mL.

Newborn	ERT Mother Treatment Status	Mother’s Lyso-Gb1 Level (ng/mL)	Newborn’s Lyso-Gb1 Level at Delivery (ng/mL)	Ratio of Mother’s Results and the Newborn Result
N1	No	577.2	77.7	7.4
N2	Yes	80.9	17.2	4.7
N3	Yes	34.1	9.8	3.5

## Data Availability

The original contributions presented in this study are included in the article. Further inquiries can be directed to the corresponding author(s).
